# The Sea as a Rich Source of Structurally Unique Glycosaminoglycans and Mimetics

**DOI:** 10.3390/microorganisms5030051

**Published:** 2017-08-28

**Authors:** Ariana A. Vasconcelos, Vitor H. Pomin

**Affiliations:** 1 Program of Glycobiology, Institute of Medical Biochemistry Leopoldo de Meis, Federal University of Rio de Janeiro, Rio de Janeiro 21941-590, Brazil; arianaavasconcelos@gmail.com; 2 University Hospital Clementino Fraga Filho, Federal University of Rio de Janeiro, Rio de Janeiro 21941-913, Brazil

**Keywords:** chondroitin sulfate, glycosaminoglycans, heparan sulfate, heparin, sulfated fucans, sulfated galactans

## Abstract

Glycosaminoglycans (GAGs) are sulfated glycans capable of regulating various biological and medical functions. Heparin, heparan sulfate, chondroitin sulfate, dermatan sulfate, keratan sulfate and hyaluronan are the principal classes of GAGs found in animals. Although GAGs are all composed of disaccharide repeating building blocks, the sulfation patterns and the composing alternating monosaccharides vary among classes. Interestingly, GAGs from marine organisms can present structures clearly distinct from terrestrial animals even considering the same class of GAG. The holothurian fucosylated chondroitin sulfate, the dermatan sulfates with distinct sulfation patterns extracted from ascidian species, the sulfated glucuronic acid-containing heparan sulfate isolated from the gastropode *Nodipecten nodosum*, and the hybrid heparin/heparan sulfate molecule obtained from the shrimp *Litopenaeus vannamei* are some typical examples. Besides being a rich source of structurally unique GAGs, the sea is also a wealthy environment of GAG-resembling sulfated glycans. Examples of these mimetics are the sulfated fucans and sulfated galactans found in brown, red and green algae, sea urchins and sea cucumbers. For adequate visualization, representations of all discussed molecules are given in both Haworth projections and 3D models.

## 1. Introduction

**Figure 1:**
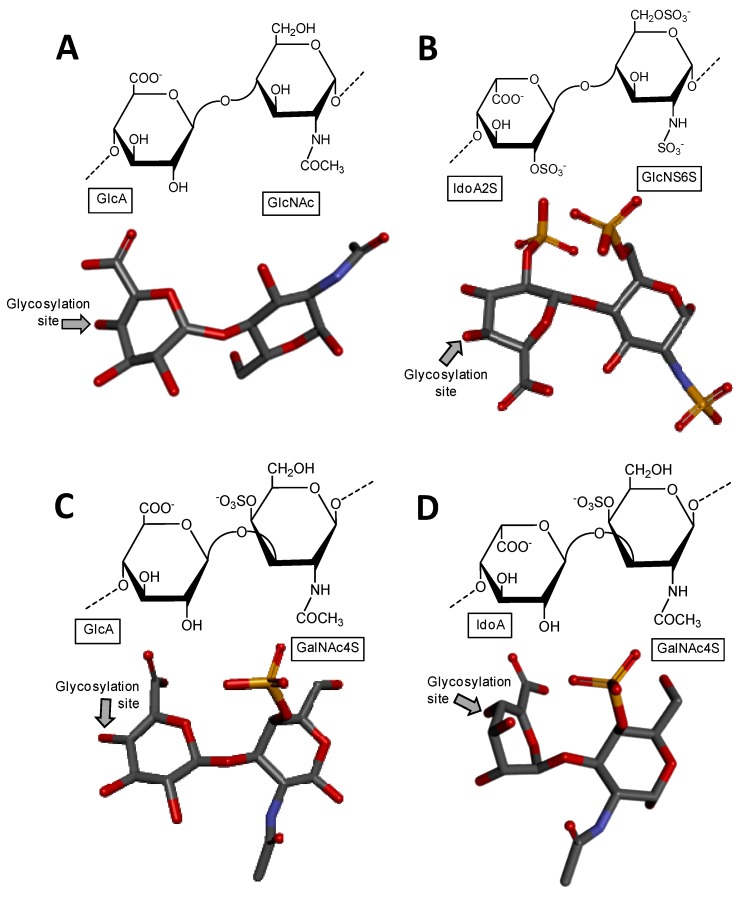

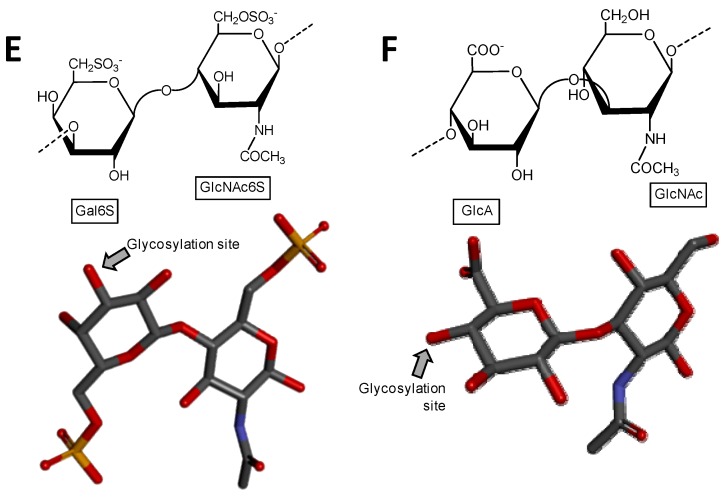
Haworth projections and stick model representations of the commonest structures in (**A**) heparan sulfate [GlcA-(β1→4)-GlcNAc] whose GlcNAc is α(1→4)-linked to the GlcA of the next disaccharide unit (extracted from PDB ID 3E7J) [35]; (**B**) heparin: [IdoA2S-(α1→4)-GlcNS6S] whose GlcNS6S is α(1→4)-linked to the IdoA2S of the next disaccharide unit (extracted from PDB ID 1HPN) [36]; (**C**) chondroitin 4-sulfate [GlcA-(β1→3)-GalNAc4S] whose GalNAc is β(1→4)-linked to the GlcA of next disaccharide unit (extracted from PDB ID 1OFM) [37]; (**D**) dermatan sulfate [IdoA-(α1→3)-GalNAc4S] whose GalNAc4S is β(1→4)-linked to the IdoA of the next disaccharide unit (extracted from PDB ID 1OFL) [37]; (**E**) keratan sulfate [Gal6S-(β1→4)-GlcNAc6S] whose GlcNAc6S is (β1→3)-linked to the Gal6S of the next disaccharide unit (extracted from PDB ID 1KES) [38]; and (**F**) hyaluronan [GlcA-(β1→3)-GlcNAc] whose GlcNAc is (β1→4)-linked to the GlcA of the next disaccharide unit (extracted from PDB ID 2BVK) [39]. The unsaturated uronic acid (∆^4,5^UroA) of the original structures 3E7J, 1OFM and 1OFL were converted, respectively, to GlcA, GlcA and IdoA. The monosaccharide nomenclatures are IdoA2S for 2-sulfated iduronic acid; GlcNS6S for *N*,6-disulfated glucosamine; GlcA for glucuronic acid; GlcNAc for *N*-acetylglucosamine; GalNAc4S for 4-sulfated *N*-acetylgalactosamine; IdoA for iduronic acid; Gal6S for 6-sulfated galactose; GlcNAc6S for 6-sulfated *N*-acetylglucosamine. The colors of the atoms used in the representations are grey for carbon, blue for nitrogen, red for oxygen and yellow for sulfur. The hydrogen atoms were omitted for visual simplification. The arrows labeled as “Glycosylation site” indicate the position of the glycosidic bond. Structures were created using ChemDraw Ultra 8.0 for Haworth projections and Discovery Studio Visualizer v.4.5 software (BIOVIA, Dassault Systèmes, San Diego, CA, USA) for 3D representations. The monosaccharides are displayed in their commonest chair configurations: ^4^*C*_1_ for GlcA, GalNAc, GlcNAc and ^1^*C*_4_ for IdoA.

Sulfated glycans are a structurally complex and widely diverse class of carbohydrates. Sulfation is a negatively charged group at neutral pH due to proton dissociation at pH 2–2.5 [1] which gives rise to the formula SO_3_^−^. This chemical group, together with some composing acidic monosaccharides, participates physicochemically to confer to sulfated glycans a strong acidic property. Hence, sulfated polysaccharides are polyanions in solution. The best known sulfated glycans are the glycosaminoglycans (GAGs). The backbone of GAGs is structurally composed of alternating hexosamine and uronic acid or galactose (Gal). The hexosamine can be glucosamine (GlcN) or *N*-acetylgalactosamine (GalNAc) and their differently substituted (sulfated) derivatives. The uronic acid can be either glucuronic acid (GlcA) or iduronic acid (IdoA). According to the kind of hexosamine and uronic acid (or Gal), different and heterogeneous chains of GAGs can be formed. GAGs are therefore classified in relation to their common structural features. Heparin, heparan sulfate, chondroitin sulfate, dermatan sulfate and keratan sulfate are all famous classes of sulfated GAGs. The structures of these sulfated GAGs and of hyaluronan (the only nonsulfated GAG type) are all described next and displayed on Figure 1.

Heparin and heparan sulfate share basically the same structural disaccharide precursor to build-up their backbones, in which different degrees of chain modification further occur according to each of these two GAG types [2,3]. The precursor is named heparosan and is structurally composed of [→4)-β-d-GlcA-(1→4)-α-d-GlcNAc-(1→] (Figure 1A), where GlcNAc stands for *N*-acetylglucosamine. Heparan sulfate is less processed by *N*-deacetylation/*N*-sulfation (which converts GlcNAc into *N*-sulfated glucosamine, GlcNS), then consequently less modified by epimerization (conversion of GlcA into IdoA) and subsequently by *O*-sulfations (at the C2 position of IdoA, and C3 and C6 positions of GlcNS). On the other hand, heparin chain suffers extensive modifications by these processes, which in turn give rises to a more variable number of structures per chain [4]. While heparin is mostly composed of [→4)-α-l-IdoA2S-(1→4)-α-d-GlcNS6S-(1→], where IdoA2S is the 2-sulfated IdoA (Figure 1B), heparan sulfate is dominantly composed of the unmodified [→4)-β-d-GlcA-(1→4)-α-d-GlcNAc-(1→] unit (Figure 1A).

Chondroitin sulfate is composed of alternating 3-linked β-d-GalNAc and 4-linked β-d-GlcA units (Figure 1C). The closely related dermatan sulfate has 4-linked α-l-iduronic acid (IdoA) instead of β-d-GlcA (Figure 1D). This happens because of C5 epimerization process during the biosynthesis of dermatan sulfate [5]. Both chondroitin sulfate and dermatan sulfate are nevertheless highly sulfated. While chondroitin sulfate can bear 4-*O*- and/or 6-*O*-sulfations at the GalNAc units (occasional sulfation can also occur at the C2 and C3 positions of GlcA), dermatan sulfate has 4-*O*-sulfation at GalNAc (Figure 1D) and occasional 2-*O*-sulfation at the IdoA unit [5]. Hyaluronan and keratan sulfate are the least processed GAG types. Keratan sulfate is composed of alternating 4-linked β-d-GlcNAc and 3-linked β-d-Gal units (Figure 1E). The *O*-sulfations can occur at the C6 positions of any monosaccharide. However, in keratan sulfate, GlcNAc units are usually more sulfated than Gal units [6] (Figure 1E). Fucosylation is an additional possible modification on chains of keratan sulfate. Hyaluronan is composed of alternating 3-linked β-d-GlcNAc and 4-linked β-d-GlcA units. No further modifications occur after the polymerization of HA, which results in a nonsulfated GAG type [7] (Figure 1F).

Because of (1) the physiological occurrence of GAGs at the extracellular matrix or cell surface, (2) their high anionic character, and (3) vast structural heterogeneity throughout their chains, GAGs are capable of interacting with various functional proteins of numerous pathophysiological systems such as wound repair [8,9], coagulation [10], thrombosis [11], cancer growth and metastasis [12,13], inflammation [14], neovascularization [15], tissue development [16], regeneration [17] and repair [18], cellular growth [19], differentiation [20] and migration [21]. As a consequence to these functions, GAGs (mainly those isolated from mammalian sources) are widely explored in medicine as therapeutics [22] or nutraceuticals [23]. However, serious downsides exist in the GAG-based therapy, especially the one based on heparin, which can lead to thrombocytopenia [24,25] and bleeding [26]. The existence of these downsides justifies the intense research on new sources of GAGs or mimetics such as (1) GAG-like molecules synthesized chemically and/or enzymatically in laboratory [27,28,29,30,31] and (2) those isolated from unique sources such as marine organisms [32,33]. Since comprehensive and updated reviews can be found in the literature regarding the GAG-like compounds synthesized by chemoenzymatic means, here we decided to focus our discussion only on some major representatives of GAGs and mimetics of marine origin. As shown for the most common GAGs discussed above, structures of the below-discussed marine GAGs and mimetics are given in both Haworth projections and 3D models. The 3D structures are derived either from the Protein Data Bank (PDB) whose coordinates are experimentally obtained from either nuclear magnetic resonance (NMR) or X-ray crystallography, or from computational simulations of molecular modeling using the MM2 force-field in the package ChemDraw Ultra 8.0 [34].

## 2. Marine GAGs

### 2.1. Holothurian Fucosylated Chondroitin Sulfate

**Figure 2:**
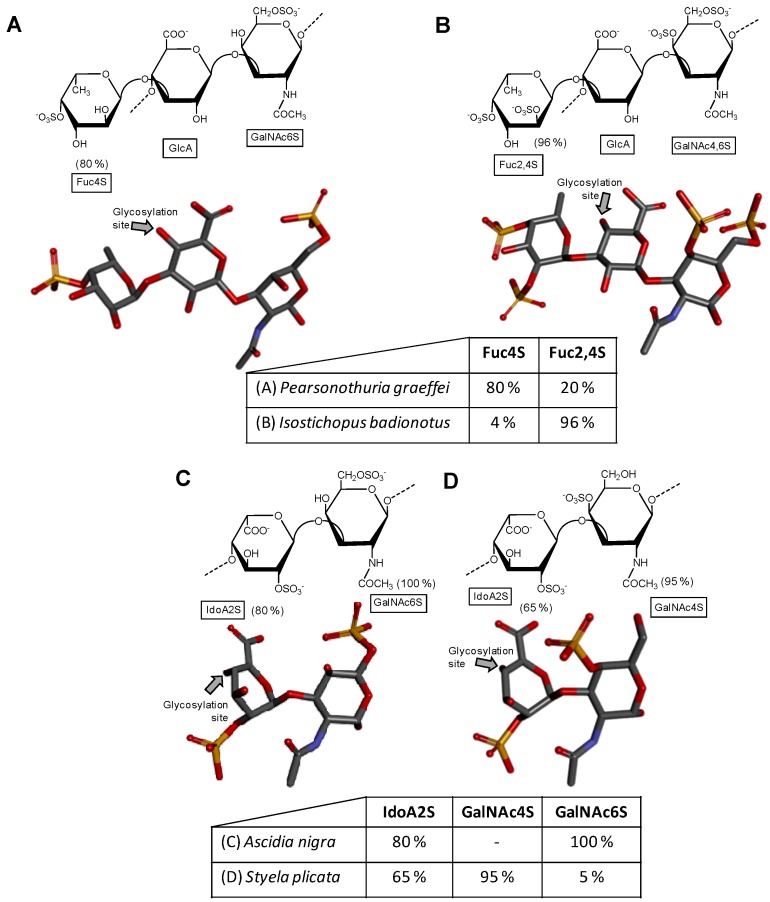
Haworth projections and stick model representations of the galactosaminoglycan structures from marine invertebrates. (**A**,**B**) Fucosylated chondroitin sulfates from two holothurian species: (**A**) *Pearsonothuria graeffei* mostly composed of {[Fuc4S-α(1→3)]-GlcA-(β1→3)-GalNAc6S} whose GalNAc6S is β(1→4)-linked to the GlcA of the next unit to make up the backbone (disaccharide of backbone extracted from PDB ID 1OFM as model) [40]; and (**B**) *Isostichopus badionotus* mostly composed of {[Fuc2,4S-α(1→3)]-GlcA-(β1→3)-GalNAc4,6S} whose GalNAc4,6S is β(1→4)-linked to the GlcA of the next unit to make up the backbone (disaccharide extracted from PDB ID 1OFM as model) [40]; Dermatan sulfate from two ascidian species: (**C**) *Ascidia nigra* mostly composed of [IdoA2S-(α1→3)-GalNAc6S] whose GalNAc6S is β(1→4)-linked to the IdoA2S of the next disaccharide unit (disaccharide extracted from PDB ID 1OFL as model) [42]; and (**D**) *Styela plicata* mostly composed of [IdoA2S-(α1→3)-GalNAc4S] whose GalNAc4S is β(1→4)-linked to the IdoA2S of the next disaccharide unit (disaccharide of backbone extracted from PDB ID 1OFL as model) [43]. The unsaturated uronic acid (∆^4,5^UroA) of the original structures 1OFM and 1OFL were converted respectively to GlcA and IdoA. The monosaccharide nomenclatures are Fuc2,4S for 2,4-di-sulfated fucose; Fuc4S for 4-sulfated fucose; GlcA for glucuronic acid; GlcNAc for *N*-acetylglucosamine; GalNAc4S for 4-sulfated *N*-acetylgalactosamine, GalNAc6S for 6-sulfated *N*-acetylgalactosamine, GalNAc4,6S for 4,6-di-sulfated *N*-acetylgalactosamine and IdoA2S for 2-sulfated iduronic acid. The colors of the atoms used in the representations are grey for carbon, blue for nitrogen, red for oxygen and yellow for sulfur. The hydrogen atoms were omitted for visual simplification. The arrows labeled as “Glycosylation site” indicate the position of the glycosidic bond. Structures were created using ChemDraw Ultra 8.0 for Haworth projections and Discovery Studio Visualizer v.4.5 software (BIOVIA, Dassault Systèmes) for 3D representations. The monosaccharides are displayed in their commonest chair configurations: ^4^*C*_1_ for GlcA and GalNAc(6S, 4,6S or 4S) and ^1^*C*_4_ for IdoA2S and Fuc(4S and 2,4S). The percentage of sulfation patterns on the lateral Fuc residues of each holothurian fucosylated chondroitin sulfate and on the IdoA and GalNAc units of the ascidian dermatan sulfates are shown as inserts at the bottom of the panels and close to the respective units.

A distinct GAG molecule from the marine environment is the fucosylated chondroitin sulfate found exclusively in sea cucumbers (Echinodermata, Holothuroidea). This GAG is composed of the regular chondroitin sulfate backbone with branches of α-l-fucose (Fuc) units 3-linked at the GlcA unit. The lateral Fuc units can show different sulfation patterns according to the holothurian species. For example, while the fucosylated chondroitin sulfate from the sea cucumber species *Pearsonothuria graeffei* is mostly 4-sulfated with minor amounts of 2,4-di-sulfation (Figure 2A) [40], *Isostichopus badionotus* synthesizes a fucosylated chondroitin sulfate almost entirely 2,4-disulfated at its branching Fuc units (Figure 2B) [40]. Different sulfation patterns can also be seen at the composing GalNAc unit of the main backbone. For example, although the GalNAc residue of *P. graffei* is mostly 6-sulfated (Figure 2A), this unit is 4,6-di-sulfated in the molecule of *I. badionotus* (Figure 2B). A comprehensive review highlighting the different structures, the potential medical properties and the scientific history of the holothurian chondroitin sulfate is available in the literature [41].

### 2.2. Tunicate Dermatan Sulfates

Although the sulfation pattern of dermatan sulfate, which is mostly composed of disaccharide building block [→4)-IdoA-(α1→3)-GalNAc4S-(β1→], can vary among tissues [44], cells [45] and pathophysiological conditions [46]; the composing GalNAc units are mostly sulfated at the C4 position (~95%) as shown in Figure 1D. But minor amounts of sulfation (~15%) can also happen at different positions such as the C6 position of GalNAc and (~10%) at the C2 position of the composing IdoA. In ascidians (Urochordata, Ascidiaceae), also known as tunicates or sea squirts, different sulfation patterns can be observed on their dermatan sulfates (Figure 2C,D) [42,43,47]. For instance, while the dermatan sulfate isolated from *Ascidia nigra* is entirely sulfated at the C6 position of GalNAc unit (100%) and mostly at the C2 position of IdoA (80%) (Figure 2C), the one isolated from *Styela plicata* is less sulfated at the C2 position of IdoA (65%) and largely sulfated at C4 position of GalNAc (Figure 2D). These are unusual sulfation patterns as compared to the typical mammalian dermatan sulfate (Figure 1D). The sulfation patterns of ascidian dermatan sulfates seem to vary according to the species of extraction. The structures of the ascidian dermatan sulfates have been characterized by NMR and disaccharide analyses [43]. This distinct sulfation patterns of the ascidian dermatan sulfates seem to play a key role in the medical properties [42,43,48]. For the best anticoagulant activity of the ascidian dermatan sulfate, the 4-*O*-sulfation of the composing GalNAc units has been reported to be essential [43].

### 2.3. Heparan Sulfate from Bivalve Nodipecten nodosum

**Figure 3:**
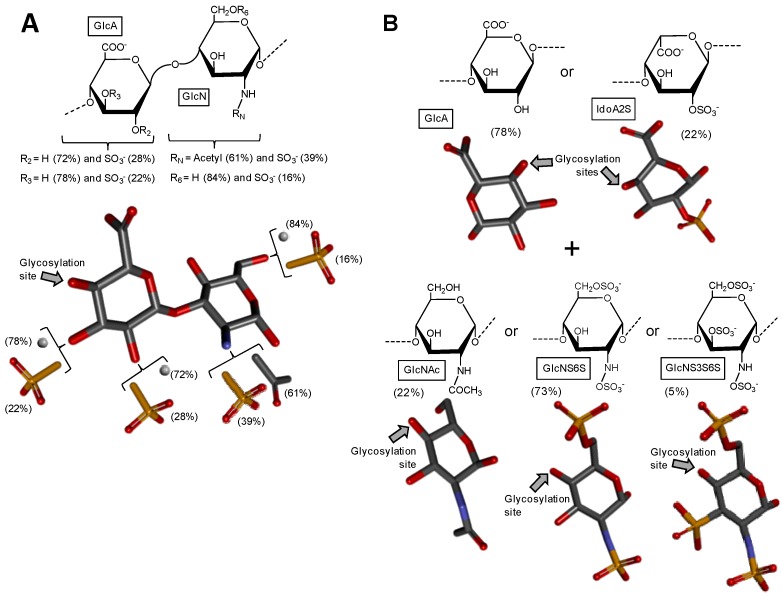
Haworth projections and stick model representations of the GAG structures from marine invertebrates. (**A**) Heparan sulfate from the bivalve *Nodipecten nodosus* composed of [GlcAR_2_R_3_-(β1→4)-GlcNR_N_R_6_] whose GlcN is β(1→4)-linked to the GlcA of the next disaccharide unit (disaccharide extracted from PDB ID 3E7J as model). R_2,_ R_3,_ R_6_ can be either hydrogen or sulfate, and R_N_ can be either acetyl or sulfate [49]; (**B**) The hybrid heparin/heparan sulfate from the shrimp *Litopenaeus vannamei* composed mostly of [GlcA-(β1→4)-GlcNS6S] and other monosaccharides whose 3D models were extracted from PDB ID 1PHN and 3E7J) [50]. Percentages of the lateral chemical groups (**A**) and composing monosaccharide types (**B**) are indicated accordingly in the panels. The unsaturated uronic acid (∆^4,5^UroA) of the original structure 3E7J was converted to GlcA. The monosaccharide nomenclatures are GlcA for glucuronic acid; GlcN for glucosamine; IdoA2S for 2-sulfated iduronic acid; GlcNAc for *N*-acetylglucosamine; GlcNS6S for *N*,6-disulfated glucosamine; and GlcNS3S6S for *N*,3,6-trisulfated glucosamine. The colors of the atoms used in the representations are grey for carbon, blue for nitrogen, red for oxygen and yellow for sulfur. The hydrogen atoms were omitted on the structures for visual simplification but are represented in light grey discs as possible substituents. The arrows labeled as “Glycosylation site” indicate the position of the glycosidic bond. Structures were created using ChemDraw Ultra 8.0 for Haworth projections and Discovery Studio Visualizer v.4.5 software (BIOVIA, Dassault Systèmes) for 3D representations. The monosaccharides are displayed in their commonest chair configurations: ^4^*C*_1_ for GlcA, GlcN, GlcNAc, GlcNS6S, GlcNS3S6S and ^1^*C*_4_ for IdoA2S.

After extensive structural characterization of the GAG from *N. nodosus* (Mollusca, Bivalvia) based on enzymatic susceptibility and NMR spectroscopy, it has been shown that this GAG is a heparan sulfate-like compound composed primarily of alternating GlcA and GlcN units [49]. Its structure is represented in Figure 3A. Although the composing units of this mollusk GAG are typical of heparan-like molecules because of the expected amounts of *N*-sulfation in GlcN units (heparan sulfate molecules present *N*-sulfation in a range of 40–60% of total GlcN units as opposed to ≥80% for heparin molecules [16]), significant amounts of C2- and C3-positioned *O*-sulfation at the GlcA units have also been identified [49]. It has been shown that GlcA units in the mollusk heparan sulfate backbone are composed of 50%, 28%, and 22% respectively of non-, 2-, and 3-sulfation as opposed to the mainly non-sulfated GlcA units in mammalian heparan sulfate molecules (Figure 1A). This mollusk GAG has shown moderate anticoagulant and antithrombotic activities. The mollusk GAG has an anticoagulant activity of 36 IU mg^−1^, 5-fold lower than porcine heparin (180 IU mg^−1^), as measured by the activated partial thromboplastin time (aPTT) assay. It also inhibits factor Xa (IC_50_ = 0.835 microg mL^−1^) and thrombin (IC_50_ = 9.3 μg mL^−1^) in the presence of antithrombin. In vivo assays have demonstrated that at the dose of 1 mg kg^−1^, the mollusk heparan sulfate inhibited thrombus growth in photochemically injured arteries. No bleeding effect, factor XIIa-mediated kallikrein activity, or toxic effect on fibroblast cells have been observed for this bivalve GAG at the antithrombotic dose [49].

### 2.4. Hybrid Heparin/Heparan Sulfate from Shrimp Litopenaeus vannamei

A unique GAG containing structural features of both heparin and heparan sulfate altogether (Figure 3B) was recently found in the head of the shrimp *L. vannamei* (Arthropoda, Crustacea, Malacostraca) [50]. The structural characterization of this hybrid GAG was achieved based on disaccharide composition analysis combined with liquid-state NMR spectroscopy. From the NMR spectra, the GAG from *L. vannamei* has been shown a large content of the unepimerized GlcA units (77.6%) and just 22.4% of the epimerized IdoA units. This dominant GlcA/IdoA ratio is typical of heparan sulfate-like molecules [50]. In addition to that, NMR-based analyses on this unique crustacean GAG has exhibited also significant amounts of *O*- and *N*-sulfated GlcN units, including the rarest 3-sulfation as commonly seen in heparin-like molecules, although in low concentration (~5%). Measurements have indicated a total of 73% of both *N*-sulfation and 6-sulfation in GlcN units and only 22% of *N*-acetylated units in this crustacean molecule. These large percentages of *N*- and 6-sulfation at GlcN and low amounts of *N*-acetylation are typical of heparin-like molecules. Hence, this unique GAG isolated from the head of the shrimp *L. vannamei* shares structural features of both heparan sulfate and heparin combined in the same molecule. It would be interesting to determine in the future if this hybrid composition occurs mostly in the same backbone or in different chains. For this, fractionation and quantification of chain populations with different GlcA and IdoA contents would be needed. In terms of biological activity, the hybrid heparan sulfate/heparin compound from the shrimp head has presented significant anticoagulant activity as seen by both aPTT and factor-Xa inhibition assays. Curiously, in contrast to mammalian heparin, the shrimp GAG displayed negligible hemorrhagic effect. These interesting findings (significant anti-Xa activity due to heparin properties together with the low bleeding effect due to the heparan sulfate structural properties) are unique to this compound and directly explained by the presence of the mixed structural features of both GAG types at the same sample [50].

## 3. Marine GAG Mimetics

### 3.1. Sulfated Fucans

Sulfated fucans are a class of sulfated glycans exclusively found in marine organisms [51]. Structurally speaking, sulfated fucans are essentially composed of α-l-Fuc units, but other sugar types such as mannose, xylose and GlcA can also occur especially at the brown alga-derived molecules [52]. Based on what has been reported so far, sulfated fucans can be found in brown algae, sea urchins and sea cucumbers. While the sulfated fucans are structural components of the cell wall and body wall, respectively, in brown algae and sea cucumbers [51], these molecules are extracellular components of the egg jelly coat of the female gamete in sea urchins In these animals, sulfated fucans are responsible to trigger the acrosome reaction, a crucial event involved in the fertilization process of these marine invertebrates [53,54]. Although sulfated fucan structures vary according to the species of occurrence, certain features are maintained among phyla.

#### 3.1.1. Sulfated Fucans from Brown Algae

**Figure 4:**
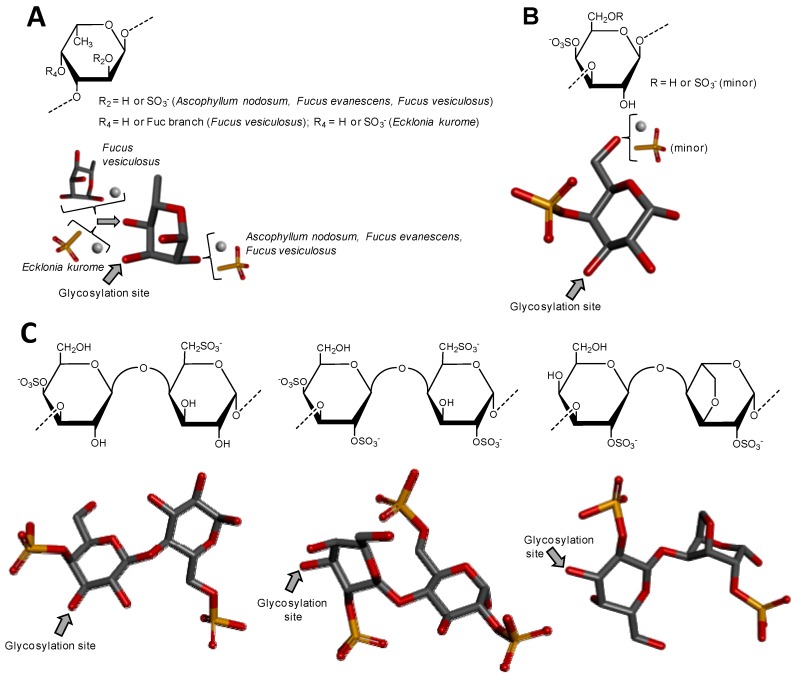
Haworth projections and stick model representations of the major components in sulfated fucans and sulfated galactans from seaweeds. (**A**) The sulfated fucans from the well-known brown alga species *Ascophyllum nodosum*, *Fucus evanescens*, *Fucus vesiculosus* and *Ecklonia kurome* are mostly composed of α(1→3)-linked α-l-Fucose (Fuc) units highly substituted by sulfation at C2 and C4 positions [55,62]. Branches of Fuc units can also occur at the C4 position through α-linkages [55,63]; (**B**) The sulfated galactan from green algae are dominantly composed of α(1→3)-linked galactose (Gal) units sulfated at position C4 [51]; (**C**) The sulfated galactans from red algae are mostly composed of regular disaccharide units alternating 3-linked β-Gal and 4-linked α-Gal residues as illustrated with the different types of carrageenans with different substitutions of sulfation and occurrence of the 3,6-anhydro-galactose units such as μ (mu) left structure, λ (lambda) middle structure and θ (theta) right structure [64]. The colors of the atoms used in the representations are grey for carbon, blue for nitrogen, red for oxygen and yellow for sulfur. The hydrogen atoms were omitted on the structures for visual simplification but are represented in light grey discs as possible substituents. The arrows labeled as “Glycosylation site” indicate the position of the glycosidic bond. Structures were created using ChemDraw Ultra 8.0 for Haworth projections and submitted to MM2 force-field. Then, Discovery Studio Visualizer v.4.5 software (BIOVIA, Dassault Systèmes) was used for 3D representations after energy minimization. The monosaccharides are displayed in their commonest chair configurations: ^1^*C*_4_ for Fuc and ^4^*C*_1_ for Gal.

Sulfated fucans from brown algae (Phaeophyceae), also known as fucoidans, are usually the most complex sulfated glycans, even though mostly composed of α-l-Fuc units (Figure 4A). The presence of other monosaccharide types associated with different and irregular sulfation patterns and occasional sparse Fuc-based branches collaborates to enhance structural complexity. The occurrence of repetitive units in sulfated fucans from brown alga is still somewhat uncertain, but evidences supporting such concept have appeared along the past years, at least for certain species [55]. This is perhaps a consequence of the improvement in instrumentation and methods designed for structural analysis of complex carbohydrates. Oligomeric repeating motifs of certain sulfated fucans of brown algae have been proposed with still high orders of structural heterogeneity [52]. Regardless of the structural patterns, sulfated fucans from brown algae are the most abundant sulfated glycans from the sea, and perhaps across the entire globe, since brown seaweeds dominate by far the sea environment in terms of both number of species (1.5 to 2 thousand) and biomass [51,55], as the sea environment totals more than 2/3 of the whole planet. Despite the great structural heterogeneity of sulfated fucans from brown algae, these molecules have been the non-GAG sulfated glycans mostly used and studied worldwide. This is likely because of their potent biomedical activities of sulfated fucans from brown algae [52,56,57] and the commercial availability of the fucoidan from *Fucus vesiculosus* [58]. Fucoidans have shown biomedical functions in multiple systems such as inflammation, coagulation, angiogenesis and cell adhesion [57]. The levels of activities in these systems are directly dependent on the structural features of the sulfated fucans [51,56,57].

#### 3.1.2. Sulfated Fucans from Invertebrates

**Figure 5:**
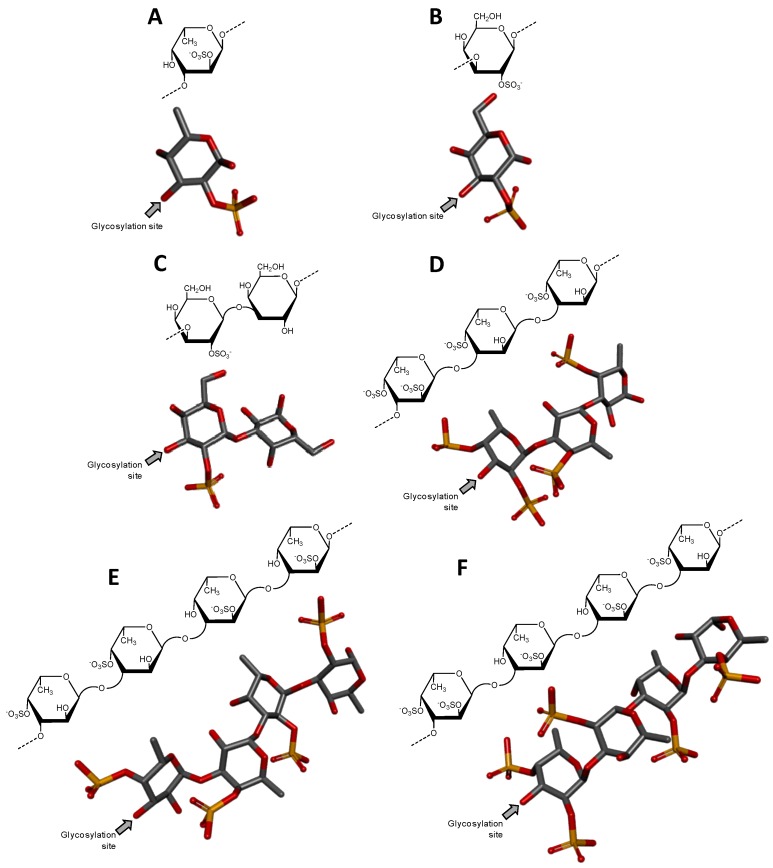
Haworth projections and stick model representations of the repetitive oligosaccharide units of sulfated fucans and sulfated galactans from echinoderms (sea urchins). While (**A**) *Strongylocentrotus franciscanus* expresses a sulfated fucan composed of 3-linked 2-sulfated α-fucose (Fuc) units [65]; (**B**) *Echinometra lucunter* synthesizes a sulfated galactan composed of 3-linked 2-sulfated α-galactose (Gal) units [66]; (**C**) The sulfated galactans isolated from *Glyptocidaris crenularis* is composed of [Gal2S-(α1→3)-Gal] whose non-sulfated Gal unit is also (α1→3)-linked to the Gal2S of the next disaccharide unit [67]; (**D**) The sulfated fucan-II from *Strongylocentrotus purpuratus* is composed of [Fuc2,4S-(α1→3)-Fuc4S-(α1→3)-Fuc4S] whose 4-sulfated Fuc unit of the reducing end is also (α1→3)-linked to the Fuc2,4S of the next trisaccharide unit [68]; (**E**) The sulfated fucan isolated from *Strongylocentrotus pallidus* is composed of [Fuc4S-(α1→3)-Fuc4S-(α1→3)-Fuc2S-(α1→3)-Fuc2S] whose 2-sulfated Fuc unit of the reducing end is also (α1→3)-linked to the Fuc4S of the next tetrasaccharide unit [69]; (**F**) The sulfated fucan isolated from *Lytechinus variegatus* is composed of [Fuc2,4S-(α1→3)-Fuc2S-(α1→3)-Fuc2S-(α1→3)-Fuc4S] whose 4-sulfated Fuc unit is also (α1→3)-linked to the Fuc2,4S of the next tetrasaccharide unit [70]. Hence, these marine sulfated glycans are regularly composed of monosaccharide (**A**,**B**); disaccharide (**C**); trisaccharide (**D**) and tetrasaccharide (**E**,**F**) building blocks. The colors of the atoms used in the representations are grey for carbon, blue for nitrogen, red for oxygen and yellow for sulfur. The hydrogen atoms were omitted for visual simplification. The arrows labeled as “Glycosylation site” indicate the position of the glycosidic bond. Structures were created using ChemDraw Ultra 8.0 for Haworth projections and submitted to MM2 force-field. Then, Discovery Studio Visualizer v.4.5 software (BIOVIA, Dassault Systèmes) was used for 3D representations after energy minimization. The monosaccharides are displayed in their commonest chair configurations: ^1^*C*_4_ for Fuc and ^4^*C*_1_ for Gal.

Sulfated fucans from marine invertebrates such as sea urchins (Echinodermata, Echinoidea) and sea cucumbers occur in oligosaccharide repetitive units with well-defined sulfation patterns (Figure 5A,D,F) and are composed solely of α-l-Fuc units. The oligosaccharide repeating building blocks can be monosaccharide as for the sulfated fucan from *Strongylocentrotus franciscanus* (Figure 5A), trisaccharide as for the second sulfated fucan from *Strongylocentrotus purpuratus* (Figure 5D) or tetrasaccharide length as for the sulfated fucans from *Strongylocentrotus pallidus* and *Lytechinus variegatus* (Figure 5E,F). Moreover, it is clear to see in Figure 5 that all structures vary in a species-specific manner. Different sulfation patterns can occur but are always restricted to C2 or C4 positions. The structures used here as representatives only have the α(1→3) type as glycosidic linkage. The molecular weight of these polymers from invertebrates are, although quite polydisperse, usually very high, frequently ranging above 100 kDa. In polymers composed of a repetitive tetrameric units, as observed for echinoderms *S*. *pallidus* and *L*. *variegatus* (Figure 5E,F), the chain extension of such glycans would range approximately over 100 tetrameric units. These sulfated fucans from marine invertebrates allow establishment of advanced structure–activity relationships, especially in terms of their anticoagulant properties [56]. In this regard, the 2,4-*O*-di-sulfation in Fuc units have been reported to be beneficial to the anticoagulant activity [56,59,60,61].

### 3.2. Sulfated Galactans

Sulfated galactans represent another class of marine GAG mimetics [64]. Like sulfated fucans, the sulfated galactans can also be found in seaweeds (green and red algae) [56] and invertebrates (sea urchins) [64,70]. And they also occur as structural components in cell walls of the algae [71] or as components of egg jelly coat of the sea urchin female gametes participating thus in the carbohydrate-mediated species-specific acrossome reaction during the initial steps of the fertilization process of these echinoderms [65,66,67]. Sulfated galactans are structurally composed of α-l-, α-d-, β-d-Gal units. Their structures vary among species, but their main structural features are still conserved among phyla as in the case of the sulfated fucans.

#### 3.2.1. Sulfated Galactans from Green Algae

Sulfated galactans usually occur in green algae (Chlorophyceae) as more homogeneous backbones than the sulfated fucans from brown algae, but still more complex than the sulfated galactans from green algae as discussed below [51,72]. Although no real evidences that regular sequences exist in sulfated galactan backbones of green seaweeds, some clues have favored the concept of chains dominantly composed of 4-sulfated 3-linked β-d-Galp units (Figure 4B) [51]. However, these chains may still bear other types of heterogeneity like pyruvylated non-reducing terminal residues and occasional Gal branches. Other sulfation positions like C6 may also occur (Figure 4B), and this collaborates to increase structural complexity to this class of polymers of green algae [51]. *Codium* has been the genus of green algae mostly studied so far in terms of sulfated galactans [73,74,75,76,77]. Although the anticoagulant activity has been the therapeutic property mostly investigated for the sulfated galactans from green algae, especially for species from the genus *Codium* [73,77,78], immunostimulating [76] and antiviral [79] activities have also been reported.

#### 3.2.2. Sulfated Galactans from Red Algae

Among the three macroalgae, red seaweeds (Rhodophyceae) are the only class able to express sulfated glycans of regular backbones [51,55]. Like GAGs, the backbones of sulfated galactans from red algae are normally composed of disaccharide repeating units, always made up of alternating 3-linked β-d-Gal and 4-linked α-d- or α-l-Gal units (Figure 4C) [68,80,81]. The possible presence of an extra-bond between C3 and C6 of the same ring leads to the 3,6-anhydro-galactose (3,6-AnGal) unit which occurs only at the 4-linked Gal unit (the right structure on Figure 4C). The enantiomeric variation, d- or l-, in this 4-linked unit, respectively, results in the terms ‘carrageenan’ and ‘agaran’. The names carrageenose or agarose are respectively related to these molecules with 3,6-AnGal units [51,64]. Sulfate esters and/or occasionally methyl esters may occur at the 2- and/or 4-position(s) of the 3-linked Gal units. These same substituents may also occur at the 2-, 3-, and/or 6-position(s) of the 4-linked Gal units. These structural variations represent the main heterogeneities in red seaweed sulfated galactans. However, since the sugar chains of these polymers are regularly composed of repeating disaccharide building blocks, the difficulties in structural characterization are significantly reduced as compared to the sulfated glycans from the other classes of macroalgae. In works concerning structural characterization of sulfated galactans from red algae, these glycans have been mostly characterized through a combination of NMR spectroscopy, particularly ^13^C-based spectra, and analyses based on chemical reactions [82,83,84,85,86]. Figure 4C shows three different carrageenan structures. Sulfated galactans of red algae have been investigated in many pathophysiological systems such as coagulation, thrombosis, microbial infections, angiogenesis and inflammation, in which the anticoagulant activity has been the most studied one [51,56,64]. Although no clear structural feature or motif of the red alga sulfate gactans have been proposed for their biomedical activities, sulfation content has been attributed to play the major role, especially in anticoagulation [59,87].

#### 3.2.3. Sulfated Galactans from Invertebrates

As discussed above for the marine invertebrate sulfated fucans, the sulfated galactans from sea urchins are composed of well-defined chemical structures such as the ones isolated and characterized for the species *Echinometra lucunter* composed of the monosaccharide repeating unit (Figure 5B) and *Glyptocidaris crenularis* composed of a disaccharide repeating unit (Figure 5C). Besides being structurally simpler (composed solely of 3-linked, and non- or 2-sulfated Gal units), sea urchin sulfated galactans seem to also occur less often than the sea urchin sulfated fucans. Like sea urchin sulfated fucans, the sulfated galactans also play a regulatory role in the acrosome reaction during the fertilization of these animals [66,67] in order to avoid cross-reaction between different species that could result into speciation [53,54]. The anticoagulant activity has been the mostly investigated biomedical function of the sulfated galactans derived from marine invertebrates [51,56]. In this regard, the 2-*O*-sulfated galactan from *E. lucunter* (Figure 5B) has been reported to be an anticoagulant glycan, while the resembling 2-*O*-sulfated fucan from *S. franciscanus* (Figure 5A), which contains all the same structural features like alpha anomericity, sulfation and linkage positions, but obviously not the same monosaccharide type, is not an anticoagulant polysaccharide [59,60,88].

## 4. Conclusions

**Figure 6:**
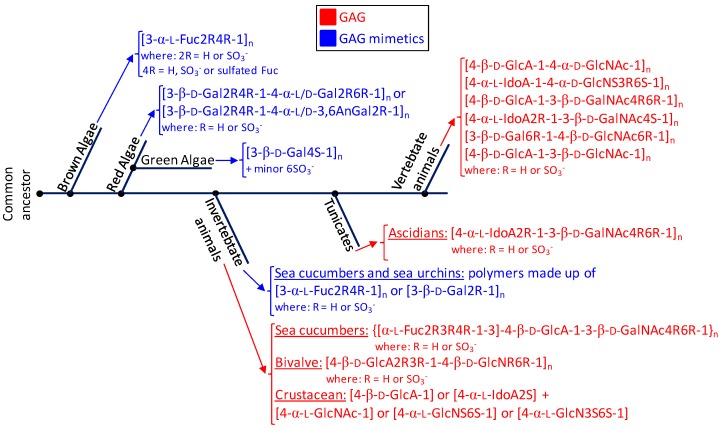
Phylogenetic relationship of glycosaminoglycans (GAGs) (red) and mimetics (blue) of seaweeds (brown, red and green algae), marine invertebrate animals, marine tunicates and vertebrate animals (from marine or terrestrial environments). The structural abbreviations are S for sulfation, R for radical, Fuc for fucose, Gal for galactose, GlcA for glucuronic acid, GlcNAc for *N*-acetylglucosamine, GalNAc for *N*-acetylgalactosamine, IdoA for iduronic acid and GlcN for glucosamine. R and S stand for radicals and sulfation, respectively. The major goal for showing this simplified scheme is to just illustrate the principal structures of the sulfated glycans expressed in the classes of organisms discussed herein and not the accurate evolutionary relationship between the cited organisms in terms of sulfated glycan-related genotypes or phenotypes or through the evolution history.

In this review, we have highlighted the unique structural features of the marine GAGs and mimetics. For this, we have started this report by introducing the major structural features of the commonest GAGs derived from mammalian sources which are largely investigated worldwide. These molecules are heparin, heparan sulfate, chondroitin sulfate, dermatan sulfate, keratan sulfate and hyaluronan. These GAGs present important medicinal application. As an alternative source to these classes of sulfated glycans, the marine organisms including macroalgae (brown, green and red algae) and invertebrates (ascidians, mollusks, crustaceans and echinoderms) are capable of synthesizing GAGs and GAG mimetics of very distinct structures than those seen from the terrestrial origins. For instance, fucosylated chondroitin sulfate can be found in the body wall of sea cucumbers. In these holothurian molecules, sulfation patterns of the Fuc branches and of the GalNAc units vary according to species. The body wall of ascidian can present uncommon dermatan sulfates whose sulfation patterns also change from species to species. The gastropode *N. nodosus* expresses a unique sulfated glucuronic acid-containing heparan sulfate in its viscera. The shrimp *L. vannamei* synthesizes a GAG type in its head with common structural features of both heparin and heparan sulfate. While brown algae express sulfated fucans with heterogeneous structures in their cell walls, the cell walls of species from green and red algae synthesize sulfated galactans more structurally homogeneous. Green algae present sulfated galactans mostly composed of 3-linked 4-sulfated Gal units. Red algae present sulfated galactans composed of disaccharide building blocks of alternating 3-linked and 4-linked Gal units. Sulfated glycans (GAGs or mimetics) exert biomedical effects in many pathophysiological systems including coagulation/thrombosis, inflammation, angiogensis, cancer growth and spread (metastasis), cell migration and differentiation, tissue development and repair, and microbial infections. The levels in their biomedical functions are dependent on their structural features, mainly sulfation patterns and content. As stated above, sulfation profiles of marine GAGs and mimetics can vary with species, but, in the mammalian systems, the sulfation patterns of GAGs may also vary with other factors such as age [89,90], tissue type [91], pathologies and levels [92]. The phenomenon of sulfation is therefore of complex nature and very sensitive to detailed sources of the compounds being examined or commented upon. Besides that, since sulfation patterns have a direct impact on the biomedical activities of the sulfated sugars, detailed comprehension on the influential mechanisms and/or factors involved in sulfation patterns is of great relevance to the science of these compounds. For proper visualization of all structures discussed in this report, representations were given in both Haworth projections and 3D models. In Figure 6, we offer a simplified phylogenetic scheme containing all sulfated glycans discussed in this report. Based on what has been discoursed in this review, the sea represents a rich source of structurally unique GAGs and mimetics. These unique marine sulfated glycans (Figure 2, Figure 3, Figure 4 and Figure 5) comprise promising alternatives to the traditional GAGs (Figure 1) in future investigations as potential carbohydrate-based models in pharmacological investigations [93,94].
